# Ubiquitin-Fold Modifier 1 Acts as a Positive Regulator of Breast Cancer

**DOI:** 10.3389/fendo.2015.00036

**Published:** 2015-03-20

**Authors:** Hee Min Yoo, Jong Ho Park, Young Joo Jeon, Chin Ha Chung

**Affiliations:** ^1^Institute for Protein Metabolism, School of Biological Sciences, Seoul National University, Seoul, South Korea

**Keywords:** ASC1, breast cancer, ERα, post-translational modification, UFM1

## Abstract

Estrogen receptor-α (ERα) is a steroid hormone-sensitive transcription factor that plays a critical role in development of breast cancer. The binding of estrogen to ERα triggers the recruitment of transcriptional co-activators as well as chromatin remodeling factors to estrogen-responsive elements (*ERE*) of ERα target genes. This process is tightly associated with post-translational modifications (PTMs) of ERα and its co-activators for promotion of transcriptional activation, which leads to proliferation of a large subset of breast tumor cells. These PTMs include phosphorylation, acetylation, methylation, and conjugation by ubiquitin and ubiquitin-like proteins. Ubiquitin-fold modifier 1 (UFM1), one of ubiquitin-like proteins, has recently been shown to be ligated to activating signal co-integrator 1 (ASC1), which acts as a transcriptional co-activator of nuclear receptors. Here, we discuss the mechanistic connection between ASC1 modification by UFM1 and ERα transactivation, and highlight how the interplay of these processes is involved in development of breast cancer. We also discuss potential use of UFM1-conjugating system as therapeutic targets against not only breast cancer but also other nuclear receptor-mediated cancers.

## Introduction

Protein modifications by ubiquitin and ubiquitin-like proteins, including SUMO and ISG15, have emerged as critical regulatory processes, such as in the control of cell cycle, stress response, signaling transduction, and immune response. Moreover, deregulation of the modification systems often gives rise to numerous human diseases, such as cancers, neurodegenerative diseases, and immune diseases (1–4).

Ubiquitin-fold modifier 1 (UFM1) is the most recently identified ubiquitin-like protein (5). Like ubiquitination, protein modification by UFM1 (ufmylation) utilizes a cascade three-enzyme system: UBA5 as an UFM1-activating E1 enzyme, UFC1 as an UFM1-conjugating E2 enzyme, and UFL1 as an UFM1 E3 ligase. This ufmylation process can be reversed by UFM1-specific proteases (UFSPs) (6). All of the proteins involved in reversible protein modification by UFM1 are conserved in metazoa and plants, but not in yeast, implicating its specific roles in multicellular organisms.

Not only estrogen receptor α (ERα) itself but also its co-regulators, including SRC1 and p300, are known to undergo a wide variety of post-translational modifications (PTMs), such as phosphorylation, acetylation, methylation, ubiquitination, and sumoylation. Moreover, these PTMs have been identified as critical events that regulate the expression of ERα and its transcriptional co-regulators and their stability, subcellular localization, and sensitivity to hormonal response (7–12). Although the components of estrogen signaling pathways are suitable and efficient targets for breast cancer therapies, the role of their PTMs in initiation and progression of breast carcinogenesis remains largely elusive.

Activating signal co-integrator 1 (ASC1), originally identified as thyroid hormone receptor interactor 4 (TRIP4), is one of ERα transcriptional co-activators (13). It also serves as a co-activator of other nuclear receptors, such as thyroid hormone receptor (TR) and retinoic acid receptor α (RARα) (14–16). However, it remained unknown whether ASC1 also undergoes PTMs and how the PTMs of ASC1 influence its co-activator function toward nuclear receptors.

In this review, we will provide an overview on current and emerging roles of the UFM1 system, with a focus on ASC1 ufmylation in regulation of breast cancer development. A thorough understanding of ASC1 ufmylation would promote not only the identification of new markers for prognosis of breast cancer but also the development of novel therapeutic strategies.

## Properties of UFM1

UFM1 consists of 85 amino acids with a predicted molecular mass of 9.1 kDa. Its gene is located in human chromosome 13q13.3. UFM1 is expressed in human cells as a precursor with a C-terminal Ser-Cys dipeptide extension, which needs to be processed by UFSPs prior to conjugation to target proteins (5). Matured UFM1 has a single glycine residue at the C-terminus, which also is required for conjugation to its target proteins, unlike ubiquitin and most other ubiquitin-like proteins, such as SUMO and NEDD8, which have a conserved C-terminal di-glycine. UFM1 is localized in both the nucleus and the cytoplasm (5).

**Figure 1:**
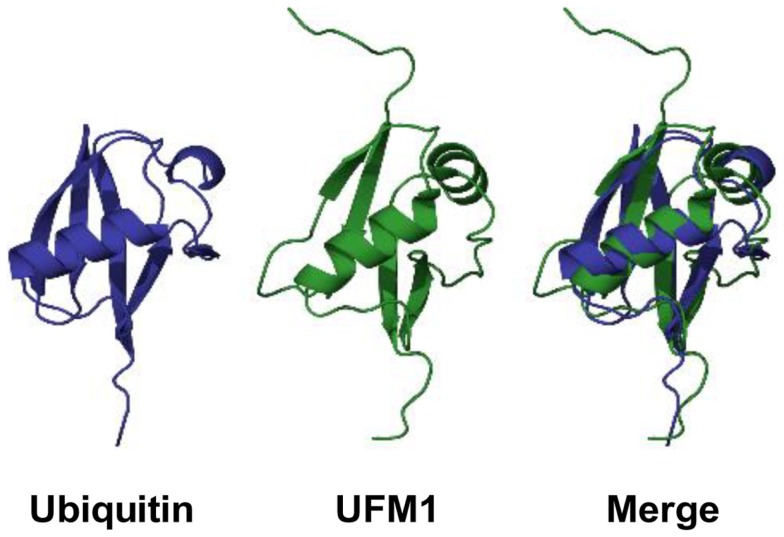
**3D structures of ubiquitin and UFM1 in human**. PDB IDs for ubiquitin and UFM1 are 1UBQ and 1WXS, respectively.

Although UFM1 has a limited amino acid sequence identity (~16%) with ubiquitin, it displays a striking similarity in its tertiary structure to ubiquitin (Figure 1). UFM1 adopts an ubiquitin-like α + β fold with ordered β−β−α−β−β−α−β secondary structure along the sequence (17). A special feature in UFM1 structure is the absence of the cluster of the acidic residues in the α1 surface, which is displayed by ubiquitin (17). Therefore, it has been suggested that UFM1 employs the uncharged surface for binding to its putative partners.

## Enzymes for UFM1 Modification

### UBA5

UBA5 (also known as UBE1DC1), an UFM1-activating E1 enzyme, consists of 404 amino acids with a predicted molecular mass of 44.7 kDa (18). Its gene is located in human chromosome 3q22.1. UBA5 is expressed in human as two distinct isoforms (amino acid sequences: 1–404 and 57–404) due to alternative splicing of its primary transcript (19, 20). The role of the additional N-terminal region (1–56) is unknown, as it is not required for UFM1 activation (19). Typically, E1 enzymes consist of the first and second catalytic cysteine half-domains (FCCH and SCCH, respectively), the adenylation domain, and the C-terminal ubiquitin-fold domain (19). UBA5 lacks the FCCH and SCCH domains, but instead simply comprises an adenylation domain, in which the catalytic cysteine (Cys250) is located, and an ubiquitin-fold domain (19, 20). Therefore, UBA5 is much smaller than other E1 enzymes, which comprise >1,000 amino acid residues. UBA5 is predominantly localized in the cytoplasm (20).

At the expense of ATP, UBA5 activates UFM1 (i.e., generates adenylated UFM1 and inorganic pyrophosphate). UFM1 is then conjugated to Cys250 of UBA5 via a thioester bond with the release of AMP (5). It has been reported that UBA5 can also activate SUMO2 under both *in vitro* and *in vivo* conditions (20). However, the loss of mouse *UBA5* has no effect on the conjugation of ubiquitin-like proteins to cellular proteins, except that of UFM1 (21). In addition, overexpression of UBA5 promotes the modification of ASC1 by UFM1, but not by any other ubiquitin-like proteins (22), indicating that UBA5 is a specific E1 enzyme for UFM1.

Significantly, UBA5-deficient mice die *in utero* due to severe anemia associated with defective differentiation of both megakaryocytes and erythrocytes, although UBA5 is dispensable for the production of erythropoietin (21). Moreover, transgenic expression of UBA5 in the erythroid lineage rescues the UBA5-deficient embryos from anemia and prolongs their survival, revealing that the UFM1-conjugating system has an essential role in erythroid differentiation. However, it is necessary to clarify whether UBA5 has other functions distinct from protein conjugation in the control of erythrocyte biogenesis in mice, as UBA4, the E1 enzyme of the Urm1 system, is known to function in tRNA uracyl thiolation in yeast, independent of protein modification by Urm1 (18).

### UFC1

UFC1 (also known as HSPC155) is an UFM1-conjugating E2 enzyme consisting of 167 amino acids with a predicted molecular mass of 19.4 kDa. Its gene is located in human chromosome 1q23.3. UFC1 is mainly localized in the nucleus and partly in the cytoplasm, but excluded from the nucleoli (http://www.proteinatlas.org). UFC1 shows low sequence homology (within a range of 13–17%) with other E2 enzymes (23). However, UFC1 has the catalytic core domain conserved in all E2-like enzymes, except that it contains an additional N-terminal helix. The active site Cys116 is located in a flexible loop that is highly solvent accessible. Upon binding of UFC1 to the ubiquitin-fold domain of UBA5, UFM1 is transferred to the cysteine residue of UFC1 by a transesterification reaction.

The neuronal cell adhesion molecule (NCAM) plays important roles in the control of cell migration, synaptogenesis, and axonal outgrowth (24). Recently, NCAM140, an isoform of NCAM, was shown to interact with UFC1 upon analysis by protein macro-array and ELISA (24). NCAM140 and UFC1 co-localize in the surface of B35 neuroblastoma cells and overexpression of UFM1 increases NCAM140 endocytosis. Therefore, UFM1 has been suggested to play a role in trafficking of cell surface molecules, although it remains unknown whether NCAM140 or other cell surface proteins are modified by UFM1.

### UFL1

UFL1 (also known as Maxer, NLBP, and RCAD) is an UFM1 E3 ligase consisting of 794 amino acids with a predicted molecular mass of 89.5 kDa. Its gene is located in human chromosome 6q16.1. UFL1 has a transmembrane domain and localizes predominantly in ER membrane. It also has a nuclear localization signal (NLS) sequence, which is functional only when the transmembrane domain is deleted (25, 26).

UFL1 does not have any domain conserved for ubiquitin E3 ligases, such as HECT, RING finger, and U-box. However, its N-terminal region (amino acid sequence: 1–202) is highly conserved across species, and sufficient for the transfer of UFM1 from UFC1 to C20orf116, the first target substrate identified for ufmylation, under both *in vitro* and *in vivo* conditions (26). Since the N-terminal region lacks the active site cysteine residue, which is typically found in HECT type E3 ligase for transthiolation reaction, UFL1 may play a role as a scaffold protein that recruits E2 enzyme and target proteins similarly to RING type ubiquitin E3 ligase.

### UFBP1

UFM1-binding protein 1 (UFBP1: also known as Dashurin and DDRGK1) consists of 314 amino acids with a predicted molecular mass of 35.6 kDa. Its gene is located in human chromosome 20q13. UFBP1 contains a transmembrane helix (amino acid sequence: 4–21), a NLS sequence (64–68), a PCI [proteasome, COP9, and initiation factor domain (228–272)], and a DDRGK sequence (253–267) (27). UFBP1 also has an N-terminal signal sequence (1–26) for its localization in ER. However, deletion of the N-terminal signal sequence leads to nuclear localization of UFBP1.

**Figure 2:**
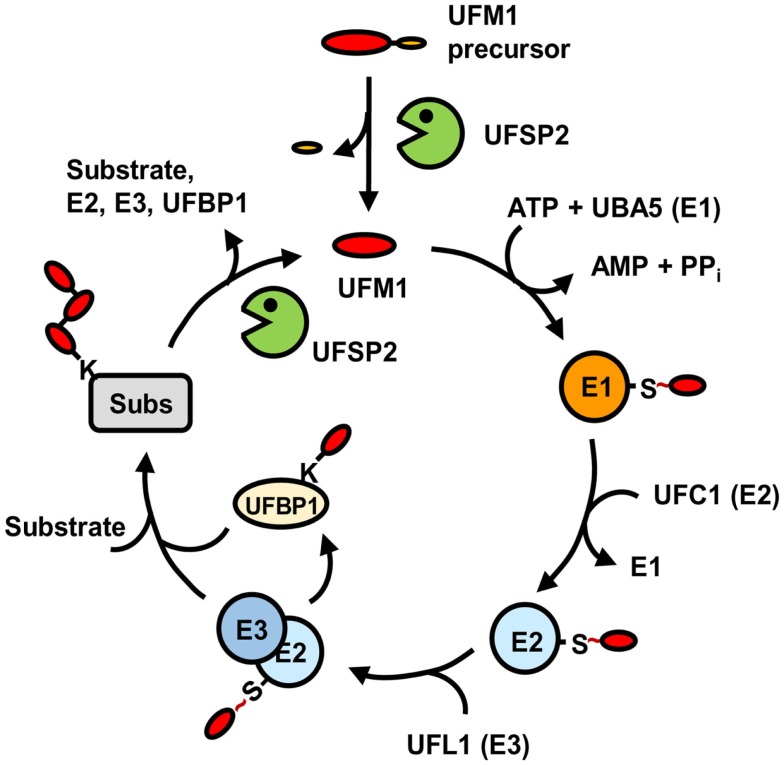
**Pathway for protein modification by UFM1**. Matured UFM1 generated from its precursor by UFSP2 is activated by UBA5 (E1), transferred to UFC1 (E2), and then conjugated to target substrates by UFL1 (E3) with the aid of UFBP1. This ufmylation pathway can be reversed by USFP2. “S ~” indicates the thioester bond.

UFBP1 was originally identified as C20orf116, which is the first target protein identified for ufmylation (26). It interacts not only with UFM1 but also with UFL1 and target proteins for ufmylation, such as ASC1 and LZAP. Depletion of UFBP1 abrogates ufmylation of the target proteins, indicating that UFBP1 serves as a cofactor as well as a substrate for ufmylation. Interestingly, ASC1 ufmylation could also be prevented by substitution of the UFM1 acceptor site Lys267 in UFBP1 with Arg. Moreover, this Lys-to-Arg mutation markedly reduces the interaction of UFBP1 with UFL1, although not with ASC1. Thus, it appears that UFBP1 may first act as a substrate of UFL1 through their weak binding and the ufmylated UFBP1, then binds to the ligase with high affinity, which might be required for the activation of UFL1. Figure 2 summarizes the overall process of protein ufmylation.

## UFM1-Specific Proteases

### UFSP1

Mouse Ufsp1 consists of 217 amino acids with a predicted molecular mass of 23.4 kDa. Like most deubiquitinating enzymes (DUBs) and ubiquitin-like protein-specific proteases (ULPs), mouse Ufsp1 belongs to the family of cysteine proteases. However, it shows no sequence homology to previously known proteases (28). This novel cysteine protease has a papain-like fold with a unique active site that is composed of a Cys box and a conserved “Asp-Pro-His” box, instead of the canonical Cys-His-Asp catalytic triad. This novel active site configuration appears to form a new subfamily of the cysteine protease superfamily (28).

**Figure 3:**
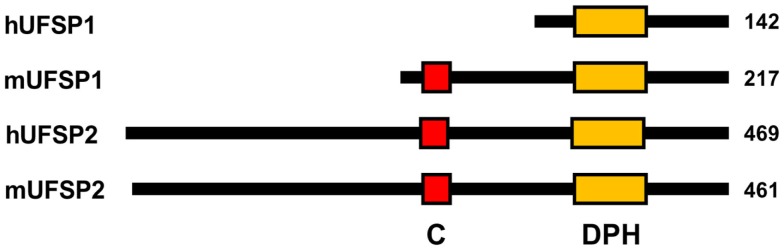
**Schematic diagram for primary structures of UFBPs**. “C” and “DPH” denote the active site Cys-box (red) and Asp-Pro-His box (orange), respectively. Note that hUFSP1 lacks the Cys box.

Human USFP1 consists of 142 amino acids with a predicted molecular mass of 15.0 kDa. Its gene is located in human chromosome 7q22.1. However, unlike the catalytically active mouse Ufsp1, human UFSP1 is expected to be non-functional, since it is shorter on the N-terminus and thereby lacks the conserved cysteine active site (Figure 3).

### UFSP2

Human USFP2 consists of 469 amino acids with a predicted molecular mass of 53.16 kDa. Its gene is located in chromosome 4q35.1. The crystal structure of mouse UFSP2 shows that the protease is composed of two domains (29). The C-terminal catalytic domain is similar to UFSP1 with the active site composed of a Cys box and a conserved Asp-Pro-His box. The novel N-terminal domain shows a unique structure and plays a role in the recognition of UFBP1. UFSP2 resides in both the nucleus and the cytoplasm. However, overexpressed N-terminal domain co-localizes with UFBP1 in ER, where UFBP1 predominantly localizes, suggesting that the N-terminal domain of UFSP2 plays an important role in the recruitment of UFBP1 to ER for reversal of ufmylation process.

A mutation within the human *UFSP2* gene has been identified in a family with an autosomal dominant form of hip dysplasia, called Beukes familial hip dysplasia (29). This mutation predicts the replacement of the highly conserved Tyr290 by His in the encoded protein. Interestingly, the substitution of Tyr282 in mouse UFSP2, which is equivalent to Tyr290 in human UFSP2, abolishes the *in vitro* UFM1-processing activity (22). Thus, it appears that impairment of reversible modification of unknown protein(s) by UFM1 is associated with an autosomal dominant form of hip dysplasia.

## Role of ASC1 Ufmylation in Breast Cancer Development

### Identification of ASC1 as a target for Ufmylation

Although UFBP1 (C20orf116) was identified as the first target protein for ufmylation (26), its biological function remained unknown. Recently, however, numerous target proteins for ufmylation have been identified by stable expression of Flag-His-UFM1 in NIH3T3 and double affinity purification using Ni^2+^-nitrilotriacetic acid-conjugated agarose and anti-Flag antibody-conjugated resins, followed by mass spectrometry (22). The identified ufmylated proteins include ASC1, a transcriptional coactivator of ERα, and LZAP (also known as CDK5RAP3 and C53) that has tumor suppressive functions, including activation of p53, induction of apoptosis, and suppression of NF-κB signaling (30–32).

All of Lys324, Lys325, Lys334, and Lys367 in ASC1 serve as the acceptor sites for UFM1 (22). Of the six lysine residues in UFM1, only Lys69 is involved in poly-UFM1 chain formation on ASC1 via isopeptide bond linkage. However, it is possible that other lysine residues may also participate in poly-UFM1 chain formation on other target proteins. Knockdown of any of UBA5 (E1), UFC1 (E2), UFL1 (E3), and UFBP1 abrogates poly-UFM1 chain formation on ASC1, indicating that UFBP1 serves as an essential cofactor for ufmylation process.

### Requirement of estrogen for ASC1 ufmylation

Endogenous ASC1 can be ufmylated upon treatment of ERα-negative cells with estrogen, but not without it (22). In the absence of estrogen, UFSP2 remains bound to the N-terminal zinc-finger domain of ASC1, and rapidly removes UFM1 molecules that are conjugated to ASC1. In the presence of the hormone, ERα forms a dimeric complex, translocates to the nucleus, and displaces UFSP2 for its binding to the zinc-finger domain of ASC1, thus allowing ASC1 ufmylation. On the other hand, no ufmylation of ASC1 can be observed in ERα-negative cells, such as MDA-MB-453, regardless of the presence of estrogen. In addition, 4-hydroxy-tamoxifen, an ERα antagonist, abrogates ASC1 ufmylation by preventing the interaction of ASC1 with ERα, indicating the requirement of estrogen binding to ERα for ASC1 ufmylation.

ASC1 acts as a general transcriptional co-activator of nuclear hormone receptors upon binding to not only ERα but also other nuclear receptors, such as androgen receptor (AR) (15). Accordingly, dihydrotestosterone (DHT), an AR agonist, induces ASC1 ufmylation in LNCap (AR-positive) cells, but not in PC3 (AR-negative) cells (22). Thus, ligand-dependent ASC1 ufmylation appears specific to cognate nuclear hormone receptors and it is likely that ligands for other nuclear receptors (e.g., all-trans-retinoic acid for RARα) can induce ASC1 ufmylation.

### Requirement of ASC1 ufmylation for ERα transactivation

The zinc-finger domain of ASC1 serves as a binding site for nuclear hormone receptors, transcriptional co-activators (e.g., SRC1 and p300), and basic transcriptional machinery (e.g., TFIIA and TBP) (14). Thus, ASC1 plays an important role as a platform that recruits the necessary components for nuclear receptor-mediated transcription. However, it remained unclear how the zinc-finger domain, a short region in ASC1 (amino acid sequence: 125-237), can simultaneously interact with such a group of the proteins. Remarkably, poly-UFM1 chain conjugated to ASC1 plays a crucial role as a scaffold protein that recruits SRC1, p300, ERα, and ASC1 itself to estrogen-responsive elements (*ERE*s) located in the promoters of ERα target genes, such as *pS2*, *Cyclin D*, and *c-MYC* (22, 33, 34). Moreover, this recruitment leads to a dramatic increase in ERα transactivation. Whereas knockdown of UFSP2 markedly promotes ERα transactivation, its overexpression abolishes it. Knockdown of UBA5 or overexpression of an ufmylation-deficient ASC1 mutant, in which the four UFM1 acceptor lysine residues are replaced by arginine, also abrogates ERα transactivation, indicating that ASC1 ufmylation is crucial for ERα transactivation.

### Promotion of tumor formation by ASC1 ufmylation

**Figure 4:**
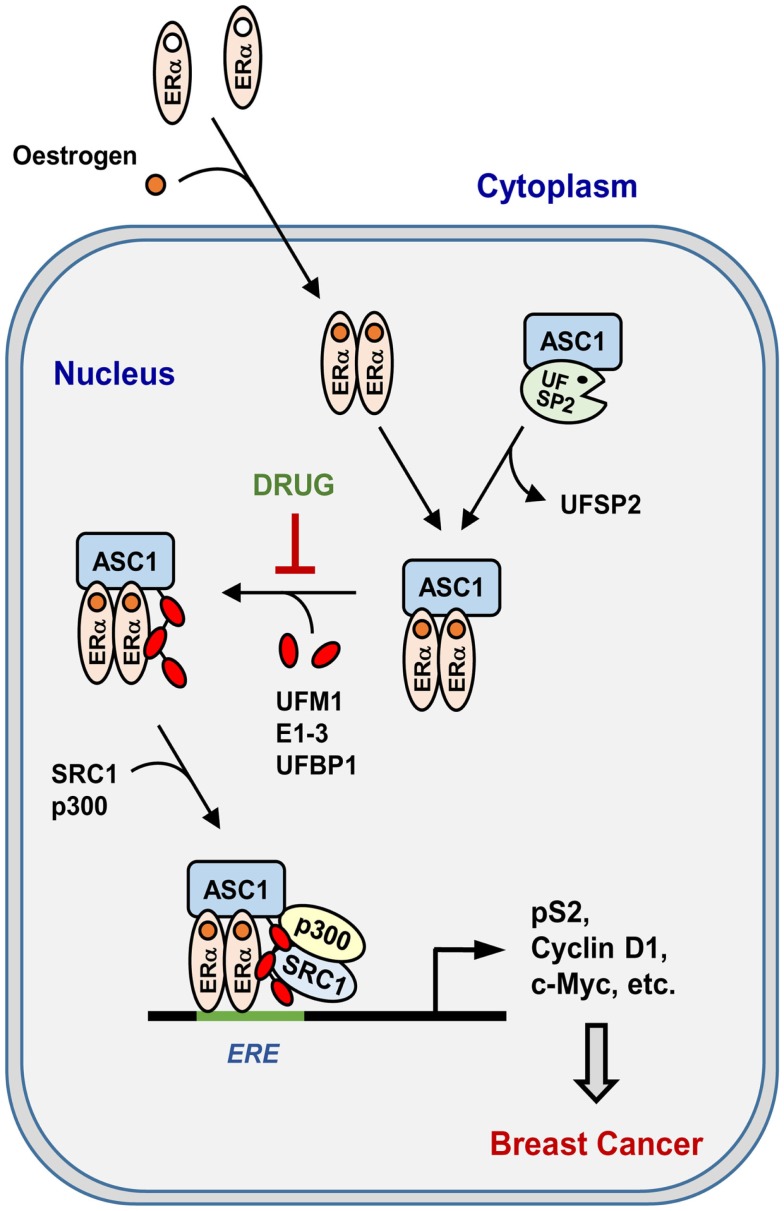
**Pathway for estrogen-induced ASC1 ufmylation for ERα transactivation**. “DRUG” indicates any small molecule that can act as a therapeutic anti-breast cancer drug by blocking ASC1 ufmylation.

Recently, colony-forming assay has shown that estrogen-induced ASC1 ufmylation is critically involved in anchorage-independent growth of ERα-positive MCF7 breast cancer cells (22). Xenograft analysis using ovariectomized mice has further revealed that ASC1 ufmylation is tightly associated with estrogen-dependent tumor formation *in vivo* (22). Whereas depletion of ASC1, UBA5, or both prevent colony formation and tumor growth, overexpression of ASC1 markedly increases them. Remarkably, depletion of UFSP2 most dramatically promotes the cell growth and tumor formation, and this promotion can be abrogated by simultaneous depletion of ASC1, implicating the role of UFSP2 as a tumor suppressor. In addition, tamoxifen could completely reverse the stimulatory effects of ASC1 overexpression and UFSP2 depletion on colony formation and tumor growth. These findings implicate a crucial role of ASC1 ufmylation in development of ERα-positive breast cancer by promoting ERα transactivity. Figure 4 summarizes estrogen-induced ASC1 ufmylation pathway for ERα transactivation, which leads to development of breast cancer.

### Possibility for development of anti-breast cancer drug

Breast cancer is one of the most prevailing cancers of woman. It is well-known that estrogen plays a critical role in the pathogenesis and development of breast cancer (35). Moreover, nearly 70% of breast cancer is ERα-positive (36). Therefore, patients with ERα-positive cancer have been treated with aromatase inhibitors, which prevent the synthesis of estrogen or with tamoxifen, which blocks the binding of estrogen to ERα (35–38). These treatments are highly effective, but many patients inevitably develop the drug-resistant invasive tumors. Therefore, new drugs against ERα-positive breast cancer are of high demand.

As to the findings that estrogen-induced ASC1 ufmylation is required for ERα transactivation and tumor formation (22), UBA5 and other components of UFM1-conjugating machinery involved in ASC1 ufmylation could be used as potential targets for development of new therapeutic drugs against ERα-positive breast cancer. Significantly, the induction of ASC1 ufmylation is not limited to estrogen, but could also occur in the presence of other ligands, such as testosterone and retinoic acid, if their cognate nuclear receptors are present in cells (22). Thus, the components of UFM1-conjugating system may also represent potent therapeutic targets in patients with other nuclear receptor-related cancers, such as prostate and leukemic cancers. Since UFSP2 knockdown leads to the most dramatic effect on the increase in cell proliferation, anchorage-independent colony formation, and tumor formation, small molecules that increase the affinity of UFSP2 to the zinc-finger domain of ASC1, or the activity of the protease could also be used as a potential drug against the nuclear receptor-mediated cancers.

## Other Biological Functions of the UFM1-Conjugating System

### UFL1 in tumorigenesis

The human *UFL1* gene is located in chromosome 6q16.1, a region that was reported to be frequently lost in prostate and gastric cancers as well as in bile duct cancer cell lines (39–41). It has also been reported that the expression of UFL1 (also called NLBP, RCAD, and Maxer) cannot be detected in invasive hepatocellular carcinoma cells including HepG2, Hep3B, HLE, and PLC, whereas it can be detected in non-invasive Huh7 hepatocellular carcinoma cell line (39). In addition, UFL1 was shown to cooperate with LZAP in suppression of cell invasion and NF-κB signaling by mutual stabilization, suggesting that UFL1 may act as a tumor suppressor (27).

However, it has also been reported that UFL1 knockdown suppresses the proliferation of C6 glioma cells and LZAP-mediated inhibition of Cyclin D1 transcription (25). In addition, UFL1 is highly expressed in human lung adenocarcinoma and its overexpression promotes the proliferation of rat H1299 lung cancer cells through interaction with p120 catenin, suggesting that UFL1 may play a role in development of lung carcinoma (42). Thus, UFL1 seems to have two opposite functions: one in tumor suppression and the other in tumor development, perhaps depending on its target proteins for ufmylation in different types of cells and tissues. In this respect, it would be of high interest to see if UFL1-mediated ufmylation differentially influences the function of LZAP and p120 catenin in the control of tumorigenesis, although it is also possible that UFL1 may regulate their functions independently of its E3 ligase activity.

Interestingly, UFBP1 was shown to bind to I-κB, stabilize it, and thereby inhibit NF-κB signaling (43). In addition, UFBP1 knockdown leads to inhibition of cell migration and invasion. Thus, UFBP1, like UFL1, plays two opposite roles as a tumor suppressor by inhibiting NF-κB signaling and as a tumor promoter by serving as a cofactor of the UFM1-conjugating system for ASC1 in development of breast cancer. UFBP1 may regulate NF-κB pathway independently of its cofactor function in ufmylation. Further studies are required to clarify the opposite dual functions of UFBP1 and UFL1 in the control of tumorigenesis and NF-κB signaling.

### The UFM1 system in ER stress response

The expression of UFM1 was shown to be up-regulated in type 2 diabetes and ischemic heart disease in mice, whose pathological conditions are associated with activation of ER stress response (44–47). ER stress induced by cyclopiazonic acid or thapsigargin, both of which are inhibitors of the ER Ca^2+^ ATPase pump, was also shown to increase the expression of UFM1, UFBP1, and UFL1 (48). Interestingly, this increase attenuates ER stress-induced apoptosis of mouse pancreatic β-cells. Recently, brefeldin, an inhibitor of vesicle trafficking, has been shown to increase the transcript level of UFM1, UFBP1, and UFL1, and this increase could not be observed in *Xbp1*^-/-^ MEFs (45). These findings suggest that the UFM1-conjugating system plays an important role in maintaining the ER homeostasis.

## Concluding Remarks

The UFM1-conjugating machinery, consisting of UBA5 (E1), UFC1 (E2), UFL1 (E3), and UFBP1, is the most recently discovered post-translational protein modification system, whose biological function is largely unknown. Intriguingly, estrogen-induced ASC1 ufmylation by this system plays a crucial role in development of breast cancer by promoting ERα-transactivation. Thus, each component of the UFM1-conjugating machinery and UFSP2 that reverses ufmylation process could be potential targets for development of drug against ERα-positive breast cancer. Since ASC1 ufmylation can be achieved by specific ligands for other nuclear receptors, such as AR and RARα, it would be of interest to examine whether ASC1 ufmylation also promotes the transcriptional activity of the nuclear receptors and thereby the receptor-mediated cancers, such as prostate and leukemic cancers, respectively. Up to present, however, the knowledge on biological function of protein ufmylation is limited to ASC1. Significantly, ER stress induces the expression of UBA5, UFBP1, and UFL1, suggesting that proteins involved in UPR response may be potential targets for ufmylation. Thus, extensive studies are of necessity to identify more target proteins for ufmylation and explore their biological function.

## Conflict of Interest Statement

The authors declare that the research was conducted in the absence of any commercial or financial relationships that could be construed as a potential conflict of interest.

## Abbreviations

AR, androgen receptor; ASC1, activating signal co-integrator 1; Cdk5rap3, CDK5 regulatory subunit-associated protein 3; DDRGK1, DDRGK domain-containing protein 1, ERα, estrogen receptor-α; ISG15, interferon-stimulated gene 15; LZAP, LXXLL/leucine zipper-containing alternative reading frame (ARF)-binding protein; Maxer, multiple α–helix protein located at ER; NCAM, neuronal cell adhesion molecule; NLBP, novel LZAP-binding protein; RARα, retinoic acid receptor α; RCAD, regulator of C53/LZAP and DDRGK1; SRC1, steroid receptor coactivator 1; SUMO, small ubiquitin-related modifier; TRIP4, thyroid hormone receptor interactor 4; UBA, ubiquitin-like modifier activating enzyme; UBE1DC1, ubiquitin activating enzyme E1-domain containing 1; UFBP1, UFM1-binding protein 1 containing a PCI domain; UFC1, ubiquitin-fold modifier-conjugating enzyme 1; UFL1, UFM1-specific ligase 1; UFM1, ubiquitin-fold modifier 1; UFSP, UFM1-specific protease; Urm1, ubiquitin-related modifier 1.
